# Efficacy and safety of acupuncture in treating post-traumatic stress disorder: A protocol for systematic review and meta-analysis

**DOI:** 10.1097/MD.0000000000020700

**Published:** 2020-06-26

**Authors:** Ning Ding, Linzhi Li, Kai Song, Ailing Huang, Hong Zhang

**Affiliations:** a Hospital of Chengdu University of Traditional Chinese Medicine; b Chengdu University of Traditional Chinese Medicine, Chengdu, China.

**Keywords:** acupuncture, protocol, post-traumatic stress disorder, systematic review

## Abstract

**Background:**

Post-traumatic stress disorder (PTSD) acts as a complex mental illness in which individuals are prone to long-lasting mental disorders after suffering traumatic events. PTSD is usually accompanied by some comorbidities, such as depressive disorder and sleep disorder, which seriously threaten patients’ life and health. Evidences showed that acupuncture could remarkably relieve the symptoms of PTSD patients. The review aims at assessing the safety and effectiveness exhibited by acupuncture for treating PTSD patients.

**Methods and analysis:**

The literature identified by searching 8 English electronic databases and 5 Chinese electronic databases from their inception to April 20, 2020 will be incorporated into the study. Two researchers will independently take charge of the research selection, the data extraction, as well as the assessment on research quality. The primary outcomes will be total PTSD symptoms, measured by different instruments including interviews and self-report measures. Data analysis will be performed via the RevMan 5 software, and Grading of Recommendations Assessment, Development, and Evaluation will help to assess the evidence level. A heterogeneity *x*^2^ test, the Higgins’ *I*^2^ test as well as visually inspecting the forest plot will help to investigate the heterogeneity of data. A sensitivity analysis and subgroup analyses will assist in investigating the sources of heterogeneity.

**Ethics and dissemination:**

The review neither assesses the individual information of patients nor impacts their rights, so it is not necessary for it to be approved by ethical institution. The article will be published in a peer-reviewed journal and present at relevant conferences.

**OSF registration number:**

https://osf.io/dc3js.

## 1 Introduction

Post-traumatic stress disorder (PTSD) acts as a complex mental illness in which individuals are prone to long-lasting mental disorders after suffering traumatic events. Its core symptoms are classified into 4 clusters:

(1) persistent event re-experiencing, for example, flashbacks and nightmares;
(2) persistent avoidance regarding trauma-related stimuli and memories;
(3) negative mood and cognitive changes, for example, a pervasive sense of imminent threat, uncontrollable thoughts and mood swings; and
(4) obvious reactivity and arousal changes, for example, panic attacks, hypervigilance, depression, and insomnia.^[1–3]^

What's more, PTSD is usually accompanied by many comorbidities, such as depressive disorder, anxiety disorder, and sleep disorder, which seriously threaten patients’ life and health.^[4]^ A study^[5]^ showed that the overall prevalence of PTSD was about 11%. PTSD annual prevalence was found to be 1.1% in transnational samples from 27 countries.^[6]^ In the United States, the incidence of PTSD is up to 3.5%, much higher than that of other mental disorders. According to a survey, many veterans were diagnosed with PTSD, and their prevalence was as high as 23%.^[7]^ At present, first-line therapies^[8]^ for PTSD include:

(1) Psychotherapy. The most common psychotherapy for PTSD treatment is cognitive-behavioral therapy, including trauma focused exposure therapy, cognitive restructuring therapy, and stress inoculation therapy, and so on;
(2) Pharmacotherapy. Only 2 kinds of medicines, namely, paroxetine and sertraline, are approved by FDA for treating PTSD.^[9]^

In addition, venlafaxine and nefazodone have also been recommended for PTSD.^[10]^ They are antidepressants in the class of selective serotonin reuptake inhibitors (SSRIs). Despite the good tolerance of SSRIs treatment, the anxiety symptoms, jitteriness, restlessness, headache or insomnia increase in the first few days or weeks following treatment and there are other side-effects including nausea, anorexia fatigue, dizziness, and weight gain, which may affect the compliance of patients.^[11]^ Currently, commonly used SSRIs class antidepressants and venlafaxine are not recommended as first-line drugs.^[12]^ Acupuncture, as a classic traditional Chinese medicine therapy, has played a full advantage in mental diseases such as depression, anxiety, and insomnia. Acupuncture is accepted by the majority of patients. Evidences showed that acupuncture could remarkably relieve the symptoms of PTSD patients.^[9,13,14]^ The study aims at assessing the treatment effectiveness and safety of acupuncture for PTSD patients.

## 2 Methods

### 2.1 Study registration

This systematic review protocol has been registered on Open Science Framework platform and the registration number is https://osf.io/dc3js.

The written document of the systematic review protocol followed the statement guidelines of preferred reporting items for systematic reviews and meta-analyses protocols.^[15]^

### 2.2 Inclusion and exclusion criteria regarding study selection

Inclusion criteria: All randomized controlled trials (RCTs) of using acupuncture to treat PTSD. The review will be presented only in Chinese and English. Exclusion criteria: Non-RCTs, quasi-RCTs, animal studies, case series, and reviews.

### 2.3 Types of participants

Participants refer to patients clinically diagnosed with PTSD. No restrictions exist regarding gender, nationality, educational background, job, and ethnicity. Participants will include, but not be limited to, combat veterans, war refugees, earthquake survivors, male and female prison inmates, socially disadvantaged persons, police officers, firefighters, as well as nurses and doctors under stress.

### 2.4 Types of interventions

#### 2.4.1 Experimental interventions

The experimental group will consist of patients receiving manual acupuncture, body acupuncture, electroacupuncture and other types of acupuncture treatment, as well as those receiving treatment combined with acupuncture.

#### 2.4.2 Control interventions

The control group will consist of patients receiving control interventions like sham acupuncture, placebo acupuncture, western medicine, routine care, herbs, conventional therapy or no treatment (waiting list control).

### 2.5 Types of outcome measures

#### 2.5.1 Primary outcomes

The primary outcomes will be total PTSD symptoms, measured by different instruments including interviews and self-report measures. Interviews will include, but not be limited to, Clinician-Administered PTSD Scale for DSM-5,^[16]^ PTSD Symptom Scale Interview (PSS-I and PSS-I-5),^[17]^ Structured Interview for PTSD,^[18]^ Structured Clinical Interview: PTSD Module,^[19]^ and Short PTSD Rating Interview.^[20]^ Self-Report Instruments will include, but not be limited to, Davidson Trauma Scale,^[21]^ Impact of Event Scale-Revised,^[22]^ Modified PTSD Symptom Scale,^[23]^ PTSD Checklist for DSM-5,^[24]^ and PTSD Symptom Scale Self-Report Version.^[25]^

#### 2.5.2 Secondary outcomes

If there are no interviews or self-report measures in the study, the descriptions of PTSD symptoms will be categorized as secondary outcomes.

## 3 Search methods for study identification

### 3.1 Electronic searches

Electronic searches will focus on databases of Nature, Science Online, PubMed, the Cochrane Library, MEDLINE, WorldSciNet, EMbase, Allied and Alternative Medicine, the Wanfang Databse, China Biology Medicine Disc, China National Knowledge Infrastructure, the Chongqing VIP, and Chinese Science and Technology Periodical Database, with the temporal from the inception of database to April 20, 2020.

### 3.2 Other search resources

Manually retrieving and reviewing a reference list of those potential and qualified studies together with relevant system reviews will help to confirm the location of other RCTs. Researchers will contact the author of trial for getting the latest clinical data for the convenience of ongoing RCTs.

### 3.3 Search strategy

Search terms will include 2 parts: PTSD (“post-traumatic stress disorder,” and “delayed psychogenic reaction”) and acupuncture (“acupuncture,” “acupuncture points,” “body acupuncture,” “manual acupuncture,” “electroacupuncture,” and “warm needle therapy”). Terms of “zhenjiu,” “zhenci,” “dianzhen,” “wenzhen,” “chuangshanghouyingjizhangai,” “chuangshanghouyalixinlizhangaizheng,” “yanchixingxinyinxingfanying,” “chuangshanghouyalifanying,” and “suijiduizhao” will be adopted from the Chinese databases. By multiple searches and proper modifications, the final search strategy of each database will be determined. Table 1 lists the search strategy specific to the PubMed database.

**Table 1:**
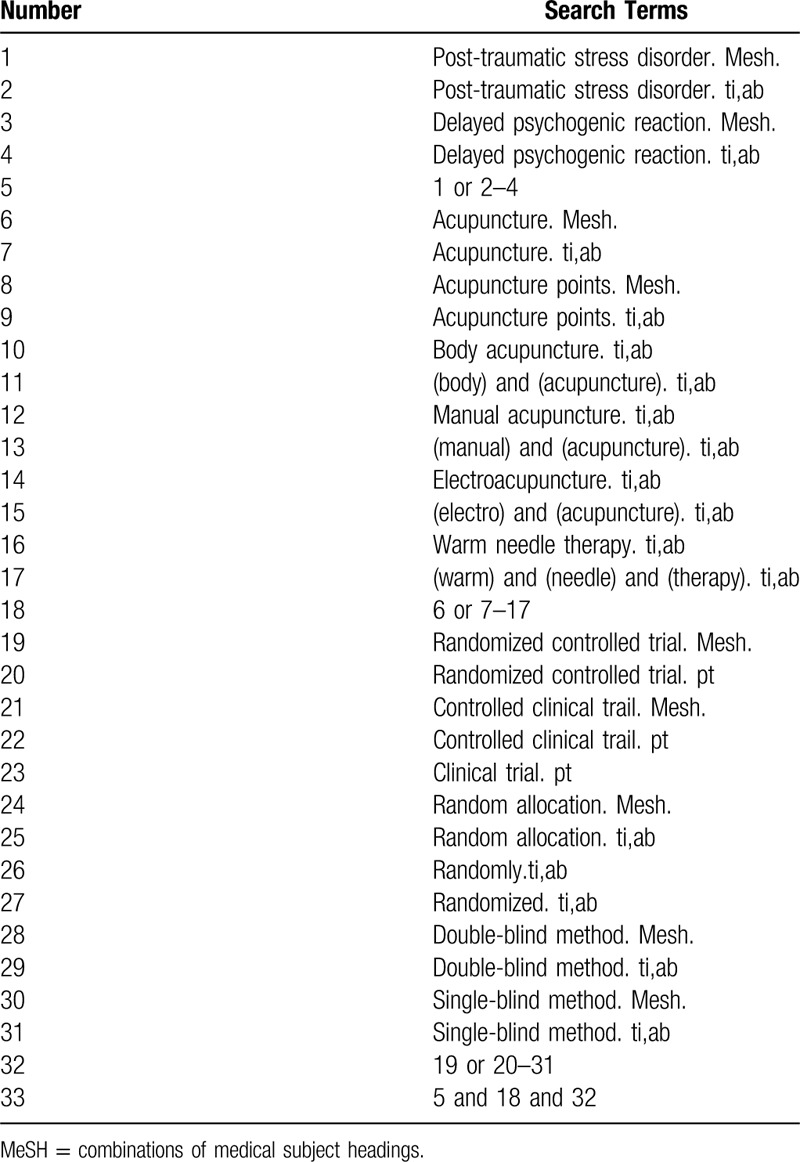
Search strategy for the PubMed database.

## 4 Data collection and analysis

### 4.1 Study selection

**Figure 1:**
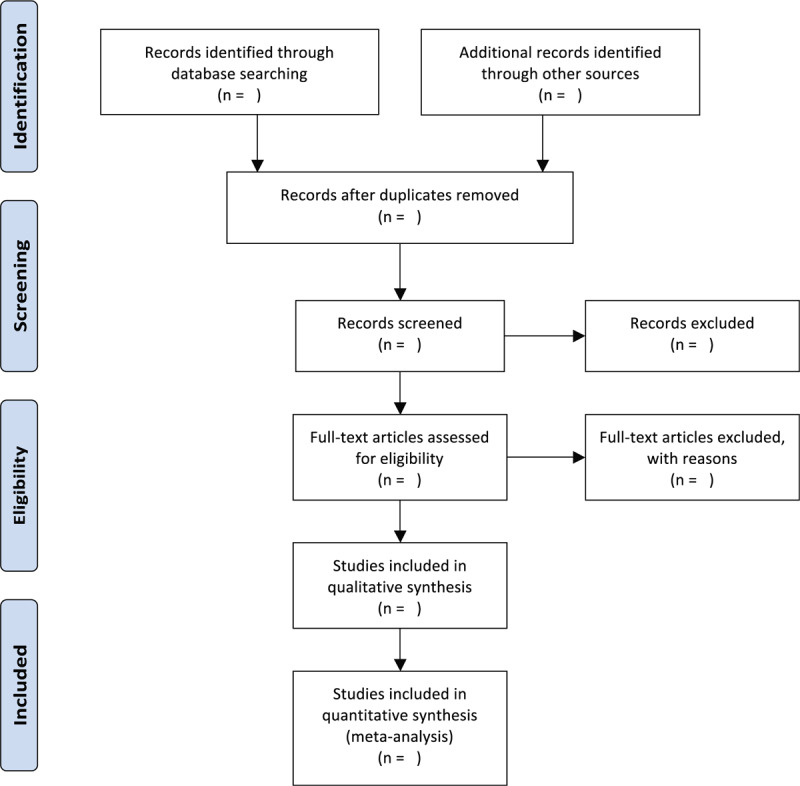
Flowchart of study selection.

After the independent information extraction from literature included in the study, 2 researchers will input the extracted information into a unified statistical table of data. Ineligible studies as well as duplicate records will be first eliminated, followed by a review on the full text of eligible studies for confirming their compliance to the abovementioned inclusion criteria. If the 2 researchers cannot come to an agreement, the final judgement will be made by a third researcher. A research flowchart will be drawn to show the whole process of study selection (Fig. 1).

### 4.2 Data extraction and management

Information of the title, the reference ID, the first author, year of publication, age of patient, intervention type, control intervention type, intervention group's sample size, intervention time, randomization, measure of outcome, allocation concealment method, blinding method, primary outcomes, follow-up duration, fund source and type, as well as a list of the standards for Reporting Interventions in controlled trials of acupuncture will be extracted from each study. Researchers will contact the author of study in the case of insufficient reported data. If negotiation cannot help to come to an agreement of the extraction of data, the final judgement will come to a third researcher.

### 4.3 Assessment of risk of bias in included studies

Two researchers will adopt the Cochrane collaboration risk-of-bias assessment for assessing the quality of literature included in the review independently, together with completing the standards for Reporting Interventions in controlled trials of acupuncture checklist.^[26]^ Assessments include selective reporting, random sequence generation, allocation concealment, incomplete outcome data, blinding as well as other possible biases. Related standards proposed in the *Cochrane Intervention System Assessment Manual* will be considered to classify risk of bias into 3 levels, low risk, high risk, and unclear risk. Discussion will be performed to resolve the discrepancy and a third research will take charge of making the final judgement if the 2 researchers cannot come into an agreement via discussion.

### 4.4 Treatment effect measures

Odds ratios and MS and standardized mean difference will assist in measuring the treatment effects specific to dichotomous outcomes and continuous outcomes, respectively. All these outcomes report 95% confidence interval (CI).

### 4.5 Missing data management

The reason for the loss of data missed in the period of data screening and extraction will be identified in the review. Corresponding author will be contacted for obtaining missing data. With missing data unable to be obtained, available data will be analyzed only and the reason and effect of such exclusion will be explained.

### 4.6 Heterogeneity assessment

Meta-analysis will be carried out with the help of a random- or fixed-effects model. *Cochrane Handbook for Systematic Reviews of Interventions* describes that either visually inspecting forest plot, a heterogeneity *x*^2^ test, or the Higgins’ *I*^2^ statistic can help to assess the heterogeneity.^[27,28]^ The data will be pooled by a fixed-effects model with *P*-value over .10 and the *I*^2^ value less than 50%, and by a random-effects model in other cases. When a set of studies exhibit an obvious heterogeneity, factors leading to the heterogeneity will be discussed, like the characteristics of patients and the variation degree in interventions. The heterogeneity will be evaluated via the subgroup analysis or the sensitivity group if applicable.

### 4.7 Reporting bias assessment

The biases of reporting will be assessed by virtue of a funnel plot if there are more than 10 trails in the meta-analysis. The asymmetry exhibited by the funnel plot will be evaluated via the Egger and Begg tests, and *P*-value < .05 means the publication bias is significant.

### 4.8 Data synthesis

Data analysis will rely on the RevMan 5 software (V. 5.3; Copenhagen: The Nordic Cochrane Centre, The Cochrane Collaboration, 2014). The heterogeneity degree will help to confirm the model type, a random-effects model or a fixed-effects model. The 2 categorical variables will adopt the index of risk ratio or odds ratio and 95% CI. Continuous variables will adopt the index of WMD or standardized mean difference and 95% CI. Meta-analysis will not be conducted if no assessment, like subgroup analysis, is able to explain the existing meaningful heterogeneity. The subgroup analysis shall carefully consider each subgroup in certain case.

### 4.9 Subgroup analysis

Subgroup analyses will consider the heterogeneity exhibited by the acupuncture type (such as manual acupuncture, body acupuncture, or electroacupuncture), the control type (placebo or sham acupuncture, no acupuncture, medical treatment or conventional therapy), the acupoint and the clinical difference.

### 4.10 Sensitivity analysis

For testing if review conclusions are robust, primary outcomes will receive a sensitivity analysis based on the criteria involving the size of sample, the quality of heterogeneity, and the statistic model (a random-effects model or a fixed-effects model).

### 4.11 Evidence quality grading

The evidence quality of obtained results will be assessed via the Grading of Recommendations Assessment, Development, and Evaluation method.^[29]^ The assessment contains risk of bias exhibited by studies, heterogeneity, evidence directness, estimate effect precision, and risk of bias of publication. We will divide evidence into 4 categories considering the level, namely high, moderate, low, and very low risk.

### 4.12 Ethics and dissemination

The results of system review will be published in journals reviewed by peers, disseminated at related conference or publications reviewed by peers. The review neither assess individual information of patients nor affect their rights, so it does not need to be approved by ethical institution.

## 5 Discussion

PTSD acts as a complex psychological disease due to patients’ being exposed to traumatic events. It is mainly characterized by persistent even re-experiencing; persistent avoidance of trauma-related stimuli and memories; negative mood and cognition changes; and obvious reactivity and arousal changes. In more than 50% of cases, patients with PTSD are usually accompanied by other comorbidities, such as anxiety and substance-use disorders.^[30]^ The persistent condition can interfere patients with relationships, ability to hold a job, the quality of life, and overall health. What's worse, a study shows that PTSD is associated with internal disease, serious disability, and premature death.^[31]^ Based on the above situation, early identifying symptoms together with the prevention and intervention programs will assist in reducing the occurrence of PTSD and other mental diseases.^[8]^ Therapies for PTSD include psychological, pharmacologic, and other alternative interventions.^[3]^ Psychotherapy and pharmacotherapy are the conventional methods for treating PTSD patients, the latter includes anxiolytic drugs and antidepressants. However, as revealed by research, the response to abovementioned therapies, and their side effects are significantly different.^[4]^ Hence, it is necessary to develop better therapies for PTSD treatment.

Traditional Chinese medicine is used to treat disease itself rather than only symptoms. Acupuncture, accepted by the majority of patients,^[32]^ offers a new option to help patients solve mental issues caused by traumatic events. There are growing clinical and experimental evidences for promoting acupuncture to be applied for different mental health disorders. A study, to evaluate if electroacupuncture is safe and efficient, includes 138 patients who had PTSD caused by earthquake. The results show that electroacupuncture is significantly effective compared with previous treatment, in addition, the electroacupuncture group exhibits a stronger efficacy relative to the paroxetine group.^[33]^ Moiraghi et al suggest that acupuncture may serve as a helpful intervention tool for public health, which could quicken the psychologic health recovery speed in disaster-stricken areas.^[2]^ According to the research, acupuncture can affect the autonomic nervous system, and the prefrontal as well as limbic brain structures, making it able to relieve the symptoms of PTSD.^[34]^

The review holds the purpose of providing extra relevant knowledge regarding the available option and clinical application to clinicians. Meanwhile, the resulting evidence of this review may contribute to essential information that can help health policy makers, practitioners, acupuncturists, and patients.

## Author contributions

**Conceptualization:** Ning Ding, Linzhi Li, Kai Song.

**Data curation:** Ning Ding, Linzhi Li, Kai Song.

**Formal analysis:** Kai Song, Ailing Huang.

**Funding acquisition:** Ning Ding, Rensong Yue.

**Methodology:** Ailing Huang.

**Project administration:** Ning Ding, Linzhi Li, Kai Song.

**Writing – original draft:** Ning Ding, Linzhi Li.

**Writing – review & editing:** Hong Zhang.

All authors read the article and approved it for publication.
